# Supplementary material for: Risk factors and predictive model construction for lower extremity arterial disease in diabetic patients
Source: PLoS One. 2024 Dec 30;19(12):e0314862. doi: 10.1371/journal.pone.0314862 (PMC11684652; doi:10.1371/journal.pone.0314862)
Supplement: S1 Table — Abbreviations: SBP, Systolic Blood Pressure; DBP, Diastolic Blood Pressure; BMI, Body Mass Index; CHD, Coronary Heart Disease; MI, Myocardial Infarction; CHF, Congestive Heart Failure; AS, Atherosclerosis; FLD, Fatty Liver Disease; CLD, Chronic Liver Disease; PCOS, Polycystic Ovary Syndrome; MEN, Multiple Endocrine Neoplasia; GLU, Glucose; GLU_2H, 2-hour Postprandial Glucose; HBA1C, Hemoglobin A1c; GSP, Glycated Serum Protein; TG, Triglycerides; TC, Total Cholesterol; HDL_C, High-Density Lipoprotein Cholesterol; LDL_C, Low-Density Lipoprotein Cholesterol; FBG, Fibrinogen; UPR_24, 24-hour Urinary Protein; BUN, Blood Urea Nitrogen; BU, Blood Urea; SCR, Serum Creatinine; UCR, Urine Creatinine; SUA, Serum Uric Acid; HB, Hemoglobin; CP, C-Peptide; INS, Insulin; PCV, Packed Cell Volume; PLT, Platelets; ESR, Erythrocyte Sedimentation Rate; TBILI, Total Bilirubin; DBILI, Direct Bilirubin; TP, Total Protein; ALB, Albumin; LDH_L, Lactate Dehydrogenase; ALT, Alanine Aminotransferase; AST, Aspartate Aminotransferase; GGT, Gamma-Glutamyl Transferase; ALP, Alkaline Phosphatase; LP_A, Lipoprotein(a); PL, Phospholipids; PT, Prothrombin Time; PTA, Prothrombin Activity; APTT, Activated Partial Thromboplastin Time; FIBRIN, Fibrinogen; ALB_CR, Albumin/Creatinine Ratio; LPS, Lipase; CA199, Cancer Antigen 19–9; CRP, C-Reactive Protein; TH2, T Helper Cell 2; IBILI, Indirect Bilirubin; GLO, Globulin. (PDF) [file pone.0314862.s001.pdf]

S1 Table. Patient Characteristics in the Dataset

Category	Feature	Data type
**General Information**	Sex, Pregnant	Categorical
	Age, Height, Weight, SBP, DBP, Heart Rate, BMI	Numerical
**Comorbidities**		
Cardiovascular and Cerebrovascular Diseases	CHD, MI, CHF, AS, Arrhythmias, Hypertention, Carotid Artery Stenosis, Cerebral Apoplexty	Categorical
Liver and Gallbladder Diseases	FLD, CLD, Cirrhosis, Biliary Tract Disease	Categorical
Kidney Diseases	Nephropathy, Renal Faliure	Categorical
Endocrine Diseases	PCOS, MEN, Endocrine Disease, Hyperlipidemia	Categorical
Tumors	Digestive Carcinoma, Urologic Neoplasms, Gynecolgical Tumor, Breast Tumor, Lung Tumor, Intracranial Tumor, Other Tumor	Categorical
Other Diseases	Nervous System Disease, Respiratory System Disease, Hematonosis, Rheumatic Immunity, Pancreatic Disease	Categorical
**Laboratory Tests**	GLU, GLU_2H, HBA1C, GSP, TG, TC, HDL_C, LDL_C, FBG, UPR_24, BU, SCR, UCR, SUA, HB, CP, INS, PCV, PLT, ESR, TBILI, DBILI, TP, ALB, LDH_L, ALT, AST, GGT, ALP, LP_A, PL, PT, PTA, APTT, FIBRIN, ALB_CR, LPS, CA199, CRP, TH2, IBILI, GLO	Numerical

Abbreviations: SBP, Systolic Blood Pressure; DBP, Diastolic Blood Pressure; BMI, Body Mass Index; CHD, Coronary Heart Disease; MI, Myocardial Infarction; CHF, Congestive Heart Failure; AS, Atherosclerosis; FLD, Fatty Liver Disease; CLD, Chronic Liver Disease; PCOS, Polycystic Ovary Syndrome; MEN, Multiple Endocrine Neoplasia; GLU, Glucose; GLU_2H, 2-hour Postprandial Glucose; HBA1C, Hemoglobin A1c; GSP, Glycated Serum Protein; TG, Triglycerides; TC, Total Cholesterol; HDL_C, High-Density Lipoprotein Cholesterol; LDL_C, Low-Density Lipoprotein Cholesterol; FBG, Fibrinogen; UPR_24, 24-hour Urinary Protein; BUN, Blood Urea Nitrogen; BU, Blood Urea; SCR, Serum Creatinine; UCR, Urine Creatinine; SUA, Serum Uric Acid; HB, Hemoglobin; CP, C-Peptide; INS, Insulin; PCV, Packed Cell Volume; PLT, Platelets; ESR, Erythrocyte Sedimentation Rate; TBILI, Total Bilirubin; DBILI, Direct Bilirubin; TP, Total Protein; ALB, Albumin; LDH_L, Lactate Dehydrogenase; ALT, Alanine Aminotransferase; AST, Aspartate Aminotransferase; GGT, Gamma-Glutamyl Transferase; ALP, Alkaline Phosphatase; LP_A, Lipoprotein(a); PL, Phospholipids; PT ,Prothrombin Time; PTA, Prothrombin Activity; APTT, Activated Partial Thromboplastin Time; FIBRIN, Fibrinogen; ALB_CR, Albumin/Creatinine Ratio; LPS, Lipase; CA199, Cancer Antigen 19-9; CRP, C-Reactive Protein; TH2, T Helper Cell 2; IBILI, Indirect Bilirubin; GLO, Globulin.
