# Supplementary material for: Risk factors and predictive model construction for lower extremity arterial disease in diabetic patients
Source: PLoS One. 2024 Dec 30;19(12):e0314862. doi: 10.1371/journal.pone.0314862 (PMC11684652; doi:10.1371/journal.pone.0314862)
Supplement: Attachment — Submitted filename: Response to Reviewers.docx [file pone.0314862.s002.docx]

PONE-D-24-26075

Risk Factors and Predictive Model Construction for Lower Extremity Arterial Disease in Diabetic Patients

PLOS ONE

Dear Editors and Reviewers,

Thank you for your letter and for the reviewers' comments concerning our manuscript entitled "Risk factors and predictive model construction for lower extremity arterial disease in diabetic patients" (ID: PONE-D-24-26075). The comments provided are valuable and very helpful for revising and improving our paper, as well as having significant guiding implications for our future research. We have carefully considered the comments and made the necessary corrections, which we hope will meet with your approval. The revisions have all been marked in the revised manuscript.

**Responding to the journal's requirements:**

We sincerely apologize for overlooking PLOS ONE's style requirements. In this revision, we have carefully followed the journal's style guidelines and made the necessary adjustments to our manuscript.

Regarding code sharing, we were unable to access the PLOS Computational Biology author guidance provided by the journal, so the code used in our study was not uploaded in this revision. Our research, which utilizes the Python-based scikit-learn package, focuses on analyzing risk factors for LEAD in diabetic patients and constructing a risk prediction model to help prevent LEAD in this population. This study is more oriented towards medical objectives rather than the improvement of machine learning code. Therefore, we are not currently eager to share our code. However, we would be happy to provide the code upon request.

We update our financial disclosure in our cover letter.

Regarding data sharing, we will update the data availability statement in this revision to comply the journal's requirements in our manuscript.

Thank you very much for your valuable feedback and assistance in improving our manuscript.

**Responds to the reviewer's comments:**

**Reviewer #1:**

**1. Response to comment: Treatment factors were excluded in the prediction model. Please clarify what treatment and also provide more detail about the rational for this decision.**

**Response:**

Thank you very much for your valuable feedback. Most diabetic patients use medications such as insulin to control their blood glucose levels, and there are significant variations in both the types and dosages of these medications. If these patients also suffer from other conditions, the treatment regimen becomes even more complex. Additionally, considering the healthcare environment in China, many patients may have already begun treatment before being admitted, but their prior treatment plans may not have been appropriate. Given these practical conditions, we decided not to include treatment factors in this study design. The main reasons are as follows:

a. Including treatment factors would inevitably make the model overly complex, requiring a sample size beyond our capacity.
b. Collecting data on patients' previous treatment plans would largely rely on the accounts of the patients and their families, who lack a professional medical background. These accounts may contain inaccuracies that could affect the experimental results.
c. We aimed to make this predictive model accessible to non-professionals, so it was important to simplify the model as much as possible without compromising its discriminative ability. This would lower the threshold for use and help more LEAD patients receive treatment earlier.
d. In the future, to enable the automated operation of this predictive model, we need to rely as much as possible on data already available in the medical system, such as diagnoses and laboratory test results. Avoiding the need for secondary data entry would enhance the model's clinical usability.
e. The effects of treatment measures are somewhat reflected in patients' various clinical indicators, so we believe that excluding treatment factors would have an acceptable impact on the model.

For these reasons, we decided not to include treatment factors in this study.

**2. Response to comment: Authors only said "missing values were handled" but no further information. How were they handled should be provided.**

**Response:**

We sincerely apologize for not providing the details on how we handled missing values. For the missing data in our study, we addressed it by removing incomplete cases. We have revised the resubmitted manuscript to include these details. Specifically, in lines 111-115, we changed "Missing values were handled before model training" to "Before training the model, we excluded incomplete cases from the dataset, resulting in the removal of 15 cases. After this preprocessing step, a total of 2,985 cases were included in the model."

**3. Response to comment: Also, testing collinearity is good. But what authors deal with collinear variables should be provided.**

**Response:**

Thank you very much for your feedback. We examined the multicollinearity of the relevant risk factors using the df.corr() method from the pandas library in Python.

**4. Response to comment: All significant predictors from univariate analysis were included in multivariate analysis. How about those non-significant in univariate analysis? If not included, will you be concerned about the potential confounding?**

**Response:**

We apologize for not providing a detailed explanation on this issue. Since the differences observed in the results of univariate analysis may not accurately reflect the effect of each factor on the outcome event, we decided to include variables with statistical significance (P < 0.05) from the univariate analysis into the multivariate analysis. Additionally, to avoid omitting important variables, we also extended the inclusion criteria to P < 0.1. This approach is commonly used in medical research, and many well-regarded studies have employed this method.

Therefore, in our study, we included factors with P < 0.1 from the univariate analysis in the multivariate analysis. To clarify this issue, we have added this explanation to the revised manuscript. In lines 107-110, we changed "All significant predictors identified in the univariate analysis were incorporated into the multivariate analysis" to "Based on the results of the univariate analysis, factors with a p-value less than 0.1 were included in the multivariate analysis." In lines 158-162, we revised "In the multivariate analysis, all variables that showed a significant association with LEADDP in the univariate analysis were included" to "In the multivariable analysis, all variables that showed a strong association with LEADDP in the univariate analysis (P < 0.1) were included."

**5. Response to comment: Please present the sample characteristic comparison between the training and testing sample.**

**Response:**

Thank you very much for your suggestion. In this study, we overlooked providing the sample characteristics for the training and testing samples. After this revision, we have included a detailed description of the sample characteristics in the text. For example, in lines 117-119, we have updated it to: "The training set included 2,089 cases, with 296 cases of LEADDP and 1,793 cases of non-LEADDP. The validation set included 896 cases, with 127 cases of LEADDP and 769 cases of non-LEADDP." To make it easier for readers to understand the sample characteristics of both the training and testing samples, we have created a table that directly presents these characteristics. Please refer to Table 1 for more details.

**Table 1.** **Characteristics of Samples in the Training and Validation Sets**

|  | NO LEADDP | LEADDP | Total |
| --- | --- | --- | --- |
| Training set | 1793 | 296 | 2089 |
| Validation set | 769 | 127 | 896 |
| Total | 2562 | 423 | 2985 |

The above content constitutes our responses to your comments and the revisions made to the manuscript based on your suggestions. We must express our gratitude to you for your valuable feedback. Your recommendations, which address aspects such as study design, data preprocessing, and statistical analysis, have significantly contributed to improving the readability of our manuscript and enhancing our research. Thank you once again.

**Reviewer #2:**

**1. Response to comment: It needs a minor revision , of the statistical methods with better description of the patients and methods.**

**Response:**

Thank you very much for your comments. We have revised the description of the statistical methods, further explained the considerations and reasons for including risk factors in our study, supplemented the criteria for including risk factors in the multivariate analysis, and provided details on the handling of missing values. We have also presented the characteristics of the training and testing samples.

In lines 79-82, we added: "Due to the complex pathogenesis of LEADDP and the unclear associated risk factors, we also included more information on comorbidities and additional laboratory indicators in order to identify new risk factors and avoid omissions" to further explain the rationale for including risk factors in this study.

The original Table 1 has been moved to the supplementary materials for easier reading.

We also supplemented the criteria for including risk factors in the multivariate analysis, stating: "Based on the results of the univariate analysis, factors with a p-value less than 0.1 were included in the multivariate analysis" (lines 109-110).

We added details on the handling of missing values, as follows: "Before training the model, we excluded incomplete cases from the dataset, resulting in the removal of 15 cases. After this preprocessing step, a total of 2,985 cases were included in the model. The collinearity of the included factors was examined" (lines 113-115).

We described the sample characteristics of the training and validation sets. You can see in lines 117-119: "The training set included 2,089 cases, with 296 cases of LEADDP and 1,793 cases of non-LEADDP. The validation set included 896 cases, with 127 cases of LEADDP and 769 cases of non-LEADDP (Table 1)." This information can be visually assessed from Table 1.

**Table 1.** **Characteristics of Samples in the Training and Validation Sets**

|  | NO LEADDP | LEADDP | Total |
| --- | --- | --- | --- |
| Training set | 1793 | 296 | 2089 |
| Validation set | 769 | 127 | 896 |
| Total | 2562 | 423 | 2985 |

**2. Response to comment: The results are not well detailed and needs more refinement.**

**Response:**

We apologize for the lack of detail in the results provided. In this revision, we have addressed your comments and further enhanced the research results. We have added the units for the risk factors included in this study, which were overlooked in the initial draft. Additionally, we have supplemented the 95% confidence intervals for the multivariate analysis results.

In line 130, we added general information about the patients, such as: "The average age was 57.8 years, with 37.5% being female."

In Table 2, we have included the units for each risk factor.

In Table 3, we have refined and completed the results of the multivariate analysis. The revised Table 3 is as follows.

**Table 3. Results of the multivariate analysis**

|  | *P* value | OR ( 95% CI ) |
| --- | --- | --- |
| Sex | 0.018 | 1.422 (1.062, 1.904) |
| AS | ＜0.001 | 19.911 (12.349, 32.103) |
| Carotid Artery Stenosis | 0.002 | 2.198 (1.345, 3.591) |
| FLD | 0.004 | 1.518 (1.140, 2.023） |
| Hematonosis | ＜0.001 | 2.094 (1.467, 2.989） |
| Endocrine Disease | ＜0.001 | 2.155 (1.623, 2.862） |
| GSP | 0.014 | 1.002 (1.000, 1.004） |

Abbreviations: AS, Atherosclerosis; FLD, Fatty Liver Disease; GSP, Glycated Serum Protein.

**3. Response to comment: The discussions are weakly describing the current literature.**

**Response:**

Thank you very much for your comments. Following your feedback, we have reorganized the manuscript and revised the discussion section.

At the beginning of the discussion, we added the following content to summarize the findings of our study: "In our study, approximately 15.9% of Chinese diabetic patients had LEAD. Male gender, atherosclerosis, carotid artery stenosis, fatty liver, hematologic diseases, other endocrine disorders, and glycated serum protein were found to be independently associated with the prevalence of LEADDP. The risk prediction model constructed using Logistic regression and MLP algorithms achieved the best performance, with moderate discriminative ability" (lines 174-179).

To more accurately explain the differences between our study and a previous study, we included additional details about the study population's regional characteristics. In lines 180-182, we modified "In our study, approximately 15.9% of Chinese diabetic patients had LEADDP, whereas another study reported a prevalence of about 4.9%" to "In our study, approximately 15.9% of Chinese diabetic patients had LEADDP, whereas another study from the southern coastal region of China reported a prevalence of about 4.9%." To explain the differences in prevalence between the two studies, we added the following content in lines 183-187: "The reason for this difference is not yet clear but may be related to regional variations. Our study data are from a medical center in northern China, where the climate is colder compared to the south. The diagnosis of LEAD primarily relies on ABI (Ankle-Brachial Index). At low temperatures, the pressure in the distal arteries of the lower limbs significantly decreases, which reduces the ABI value [11]." Additionally, in lines 189-194, we added: "Furthermore, there are significant differences in dietary habits, lifestyles, and economic levels among people in different provinces of China, which may, to some extent, affect the development of diabetes and its complications. Existing research data indicate that the prevalence of diabetes varies across different provinces in China [12]. However, there have been no epidemiological reports specifically on LEADDP in China." These modifications help clarify the differences in prevalence between our study and the previous research on LEAD among Chinese diabetic patients.

In lines 199-201, we revised "However, in our study, we found that the incidence of LEADDP was higher in males" to "However, in our study, we found that there were more men than women among diabetic patients with concurrent LEAD." In line 202, we added the following content and reference: "A study on LEAD in the diabetic population also reached similar conclusions [16]." These changes help explain the gender differences in LEAD prevalence among diabetic versus non-diabetic patients.

In lines 216-218, we added: "However, in our study, there were no significant differences in the prevalence of complications such as coronary heart disease, myocardial infarction, and chronic heart failure," to further discuss our results.

To explain the mechanism by which hyperglycemia damages blood vessels, we added in lines 224-226: "Hyperglycemia promotes oxidative stress, glycoxidation, and systemic inflammation, damaging the endothelial cells of the arterial wall, leading to lipid deposition and the development of atherosclerosis [21]." To clarify the relationship between blood glucose control and LEAD risk in our study, we included in lines 231-234: "In our study, LEAD patients had poorer blood glucose control compared to those without LEAD. Although fasting blood glucose levels did not show significant differences, the postprandial 2-hour blood glucose levels were significantly elevated." To explain our findings, we added in lines 236-238: "Studies have also shown that arterial vascular damage can begin before blood glucose levels increase, with elevated insulin levels potentially being a contributing factor [21]."

To clarify our results regarding lipids, fatty liver, and BMI, we added in lines 252-254: "Elevated low-density lipoprotein levels indicate dyslipidemia. In our study, patients with both LEAD and fatty liver had an increased risk of LEAD, and the difference in BMI was not significant." In lines 258-259, we added: "This suggests that obesity may not be a direct risk factor for LEADDP, but rather an indicator of dyslipidemia."

To enhance the logical flow of the discussion, we moved the statement "Currently, no studies have analyzed the correlation between hematologic diseases and LEADDP" from lines 276-277 to lines 282-283. At the end of this section, we added: "This finding opens new avenues for research into LEADDP" (lines 283-284).

To further describe the risk prediction model, we added in lines 301-304: "This model is built using easily collectable objective clinical data, and it can be directly integrated with data from medical systems without the need for secondary collection. This allows for improved LEAD risk prediction without increasing the workload of medical personnel."

At the end of the discussion section, we added a summary of the strengths and limitations of our study. The content is as follows:"This study identified risk factors for LEAD in the Chinese diabetic population, further explored the mechanisms related to LEADDP, and developed a relatively user-friendly risk prediction model using machine learning algorithms. The model can assist specialists in identifying high-risk LEADDP patients. The limitations of this study include the following. First, the data used in this study were obtained from the China National Population Health Science Data Center, and while the data were collected by reliable medical centers, the comorbidities in the dataset were not clearly defined, which may pose limitations for broader application. Second, for the sake of model usability, this study did not include treatment factors, so the potential effects of medications on the outcomes have not been explored. Third, as this is a cross-sectional study, the research design does not allow us to establish causal relationships between the identified risk factors. Finally, the data primarily come from northern China, which may limit its generalizability to other regions." (Lines 305-317)

We hope these additions and modifications to the discussion section better describe our research.

Here, we would like to thank you again for your comments. Your feedback has summarized the issues in our study and has been very guiding for our revisions.

**Reviewer #3:**

**1. Response to comment: Please make a brief introduction and clarification of the dataset and study design the authors used. It seems to be a cross-sectional design. If so, it is inappropriate to call it incidence.**

**Response:**

Thank you for your comments. This study is a single-center, cross-sectional design, and the dataset used, the Diabetes Complications Warning Dataset, comes from the China National Population Health Science Data Center. This dataset includes general information, comorbidities, laboratory results, and other data for diabetic patients. We used univariate and multivariate analyses to identify risk factors for diabetic lower extremity arterial disease (LEADDP) and developed a risk prediction model to improve the diagnostic rate of LEADDP and enable early treatment for patients.

We apologize for the incorrect terminology used in the manuscript due to the lack of a clear definition for prevalence. Thank you for your feedback. We have revised the inappropriate parts. We changed "The study population consisted of 3,000 patients, with 476 (15.9%) developing LEAD" to "The study population consisted of 3,000 patients, with 476 (15.9%) having LEAD" (line 31-32). We revised "The primary aim of this study is to describe the incidence of LEAD in diabetic patients and to identify potential risk factors" to "The primary aim of this study is to describe the prevalence of LEAD in diabetic patients and to identify potential risk factors" (line 68-69). We changed "Significant differences were observed between patients who developed LEADDP and those who did not in terms of general information, vital signs, laboratory tests, and comorbidities (Table 2)" to "Significant differences were observed between patients who had LEADDP and those who did not in terms of general information, vital signs, laboratory tests, and comorbidities (Table 2)" (line 130-133). We revised "However, in our study, we found that the incidence of LEADDP was higher in males" to "However, in our study, we found that there were more men than women among diabetic patients with concurrent LEAD" (line 199-202). Finally, we changed "In our study, individuals with LEADDP exhibited a higher incidence of kidney disease and significantly reduced kidney function compared to those without LEAD" to "In our study, individuals with LEADDP exhibited a higher prevalence of kidney disease and significantly reduced kidney function compared to those without LEADDP" (line 262-266).

**2. Response to comment: Table 1 should be included into supplementary.**

**Response:**

Thank you for your comments. We have included Table 1 in the supplementary materials.

**3. Response to comment: The figures are very vague in the combined PDF. After downloading the original figures, it's hard to see the words alongside the bars. Please add figure legend.**

**Response:**

We apologize for not providing sufficiently clear figures earlier. In this revision, we have redrawn the figure and checked it according to the PLOS ONE requirements. You should now be able to view the content of the figure clearly in the PDF. Based on your feedback, we have added a legend to the figure. The legend is as follows:

Fig.1. Heatmap of Variable Correlations. Dark blue areas indicate strong negative correlations, while dark red areas indicate strong positive correlations. Light colors show weaker correlations. Abbreviations: AS, Atherosclerosis; FLD, Fatty Liver Disease; HBA1C, Hemoglobin A1c; TP, Total Protein. (line 122-125).

The regenerated figure is as follows.

**4. Response to comment: BP high and BP low, should be changed into systolic blood pressure and diastolic blood pressure.**

**Response:**

Thank you for your feedback. We have revised the manuscript by changing "Bp High" to "Systolic Blood Pressure" and "Bp Low" to "Diastolic Blood Pressure."

**5. Response to comment: No unit is indicated in Table 2.**

**Response:**

Thank you very much for your feedback. In this revision, we have added units to Table 2. Since the table is quite long, we have not inserted it here; you can find the revised table starting from line 134 in the updated manuscript.

**6. Response to comment: Why listing so many variables in the table 2? Are these variables all associated with LEAD? It's suggested to make clear the clinical significance of these variables with LEAD first before doing the analyses.**

**Response:**

Thank you for your valuable feedback. LEAD is a type of generalized vascular disease with a complex pathological mechanism. While past studies have provided some insights into the relevant mechanisms, differing conclusions have emerged across different studies. Therefore, we opted to include more influencing factors in our analysis to identify risk factors associated with LEAD and avoid omissions. We apologize for not clarifying these considerations in the original manuscript. Consequently, we have added the following description in lines 79-82: "Due to the complex pathogenesis of LEADDP and the unclear associated risk factors, we also included more information on comorbidities and additional laboratory indicators in order to identify new risk factors and avoid omissions."

**7. Response to comment: In the first part of the discussion, it's suggested to make a brief introduction of the main findings first. Strengths and limitation should be clarified in the discussion part also.**

**Response:**

Thank you for your feedback. In the first part of the discussion, we briefly introduce the main findings, which you can find in lines 174-179. The content is as follows:

"In our study, approximately 15.9% of Chinese diabetic patients had LEAD. Male gender, atherosclerosis, carotid artery stenosis, fatty liver, hematologic diseases, other endocrine disorders, and glycated serum protein were found to be independently associated with the prevalence of LEADDP. The risk prediction model constructed using Logistic regression and MLP algorithms achieved the best performance, with moderate discriminative ability."

At the end of the discussion, we outline the strengths and limitations of our study, which you can find in lines 305-317. The content is as follows:

"This study identified risk factors for LEAD in the Chinese diabetic population, further explored the mechanisms related to LEADDP, and developed a relatively user-friendly risk prediction model using machine learning algorithms. The model can assist specialists in identifying high-risk LEADDP patients. The limitations of this study include the following. First, the data used in this study were obtained from the China National Population Health Science Data Center, and while the data were collected by reliable medical centers, the comorbidities in the dataset were not clearly defined, which may pose limitations for broader application. Second, for the sake of model usability, this study did not include treatment factors, so the potential effects of medications on the outcomes have not been explored. Third, as this is a cross-sectional study, the research design does not allow us to establish causal relationships between the identified risk factors. Finally, the data primarily come from northern China, which may limit its generalizability to other regions."

**8. Response to comment: The key finding is the prediction model in this study. So, please clarify the main findings compared with other studies instead of talking about each risk factor in the model.**

**Response:**

Thank you for your suggestion. We would like to provide further clarification on this issue. The prediction model is one of the key findings of our study. To our knowledge, there are currently no studies that have constructed a prediction model for LEADDP using machine learning algorithms. We have made a preliminary attempt in this direction and developed a model with moderate discriminative performance, which is one of the outcomes of this research.

Additionally, our study has analyzed risk factors related to LEADDP in the Chinese diabetic population and yielded some new insights. For example, "The gender distribution of LEAD among the diabetic population is different." "We found an association between hematologic diseases and LEAD, which is less commonly discussed in published research." We believe these findings are significant as they may help us further understand the pathogenesis of LEADDP.

**9. Response to comment: It's suggested to reorganize the manuscript to highlight the main findings from this study.**

**Response:**

Thank you very much for your feedback, which is crucial for improving the readability of the paper. Based on your suggestions, we have reorganized the manuscript.

In the discussion section, we emphasized some of our significant research findings. For instance, in lines 283-284, we added "This finding opens new avenues for research into LEADDP" to highlight the potential significance of this result. In lines 301-304, we emphasized some advantages of the risk prediction model with the following content: "This model is built using easily collectable objective clinical data, and it can be directly integrated with data from medical systems without the need for secondary collection. This allows for improved LEAD risk prediction without increasing the workload of medical personnel."

These are our responses to your comments and the modifications made based on your suggestions. We would like to thank you once again. Your feedback was very thorough and detailed, and it has played a crucial role in refining this paper.

**Reviewer #4:**

**1. Response to comment: In Table 1, you present the characteristics of all patients included in the dataset. However, it is unclear who is responsible for collecting this data and by what methods. It is essential to provide more detailed information about the Diabetes Complications Warning Dataset. Specifically, who is responsible for validating the accuracy of the data?**

**Response:**

Thank you for your feedback. The dataset used in this study, the Diabetes Complications Warning Dataset, comes from the China National Population Health Science Data Center. Data collection and processing for this dataset were conducted by the General Hospital of the People's Liberation Army, one of China's top medical centers located in Beijing, a northern city in China. This institution ensures that the medical instruments used for testing are precise and accurate, holding medical device registration certificates. The testing methods are scientifically sound, and the testing processes follow standardized operational protocols. Therefore, we have confidence in the accuracy of the data in this dataset.

**2. Response to comment: Several parameters listed in Table 1 (e.g., arterial hypertension, renal failure, fatty liver, etc.) have definitions established by published standards. How were these parameters defined within your dataset? In the absence of such definitions, how can you ensure consistency in the diagnosis of conditions like renal failure across different patients?**

**Response:**

Thank you very much for your valuable feedback. We regret that the diagnostic criteria you mentioned are not explicitly defined in the dataset. We trust in the authority and professionalism of the data provider, and thus believe that there is consistency in the diagnosis of diseases such as renal failure among different patients in this dataset. However, if we expand our research to include data from other centers, particularly those that may not have the same level of authority, we cannot ensure consistency in diagnoses across different centers. We appreciate your feedback, but unfortunately, we are unable to address this issue at the moment.

**3. Response to comment: Could you elaborate on the rationale behind choosing a 7:3 ratio for the training and validation sets?**

**Response:**

Thank you for your feedback. We are happy to explain the rationale behind our choice. In machine learning, the training set is used to train the model, while the validation set is used to evaluate the model's generalization ability. When the dataset size is 10,000 or fewer, it is common practice to split the data into training and validation sets with a 7:3 ratio. This approach is widely used in machine learning engineering as it provides a good balance between training and validation. It ensures that there is enough data for model training while also providing a sufficient amount of test data for reliable evaluation. Therefore, we selected a 7:3 ratio for the training and validation sets in our study.

**4. Response to comment: Your statement regarding atmospheric changes appears to be contentious. The paper you cite associates atmospheric temperature with acute limb ischemia, rather than LEAD. How does your paper's definition of LEAD compare with the definition used in the study by Chen et al.?**

**Response:**

Thank you very much for your suggestion. We revisited the study by Chen et al., and found that it was an inappropriate citation. Although Acute Leg Ischemia and Lower Extremity Arterial Disease both involve ischemic conditions of the distal arteries, their pathophysiological mechanisms differ. We initially cited Chen et al. to try to explain the higher prevalence of LEADDP in our study. We have not found evidence of prevalence differences among LEAD patients in the same region with varying temperatures in existing research. Even in global prevalence analyses, differences between latitudes have not been clearly established. Clinically, there is a noticeable increase in LEAD patients during colder weather. Some studies suggest that lower temperatures lead to decreased pressure in the toe arteries, indicating that temperature may affect the prevalence and diagnosis of LEAD. We have revised the inappropriate parts in the manuscript. In lines 180-194, we removed the inappropriate sections and added new discussion. The revised content is as follows:

"In our study, approximately 15.9% of Chinese diabetic patients had LEADDP, whereas another study from the southern coastal region of China reported a prevalence of about 4.9% [10]. The reason for this difference is not yet clear but may be related to regional variations. Our study data are from a medical center in northern China, where the climate is colder compared to the south. The diagnosis of LEAD primarily relies on ABI (Ankle-Brachial Index). At low temperatures, the pressure in the distal arteries of the lower limbs significantly decreases, which reduces the ABI value [11]. Therefore, we believe this difference may be due to the influence of different climates in the regions where the studies were conducted. Additionally, there are significant differences in dietary habits, lifestyles, and economic levels among people in different provinces of China, which may, to some extent, affect the development of diabetes and its complications. Existing research data indicate that the prevalence of diabetes varies across different provinces in China [12]. However, there have been no epidemiological reports specifically on LEADDP in China."

We sincerely appreciate your highly professional suggestions, which have been instrumental in improving our research.

**Other changes:**

1. In lines 31-32, we changed "The study population consisted of 3,000 patients, with 476 (15.9%) developing LEAD." to "The study population consisted of 3,000 patients, with 476 (15.9%) having LEAD."

2. In lines 38-41, we changed "Our study helps identify high-risk populations and improve lower extremity health in diabetic patients." to "Our study identified the risk factors for LEAD in Chinese diabetic patients, and the constructed risk prediction model can aid in the diagnosis of LEAD."

3. In line 208, we deleted the word "therefore."

4. In lines 239-241, we deleted the sentence "Our study results indicate that glycemic-related indicators are statistically significant, but this difference was excluded in the multivariate analysis."

5. In lines 320-321, we added: "These findings contribute to a deeper understanding of the pathogenesis of LEADDP."

Thank you to the editor and reviewers for their valuable and helpful suggestions on our paper. Based on these suggestions, we have made revisions and additions to the manuscript. All the revisions have been listed in this document and marked in the revised manuscript.

Once again, we sincerely appreciate the editor and reviewers for their hard work and thorough review.
