# Supplementary material for: Risk factors and predictive model construction for lower extremity arterial disease in diabetic patients
Source: PLoS One. 2024 Dec 30;19(12):e0314862. doi: 10.1371/journal.pone.0314862 (PMC11684652; doi:10.1371/journal.pone.0314862)
Supplement: Attachment — Submitted filename: Response to Reviewers.docx [file pone.0314862.s003.docx]

PONE-D-24-26075

Risk Factors and Predictive Model Construction for Lower Extremity Arterial Disease in Diabetic Patients

PLOS ONE

Dear Editors and Reviewers,

Thank you for your letter and for the reviewers' comments concerning our manuscript entitled "Risk factors and predictive model construction for lower extremity arterial disease in diabetic patients" (ID: PONE-D-24-26075). We have carefully considered these comments, and below are our responses.

**Responding to the journal's requirements:**

Thank you for your reminder; we have reviewed the reference list again.

**Responds to the reviewer's comments:**

**Reviewer #3:**

Thank you very much for your comments. Your feedback is thorough and rigorous, which has been very helpful to us. In response to your remarks, we would like to address the following points.

**Significance and Public Health Value of the Study**

This study aims to predict the risk of LEAD in diabetic patients and to improve the diagnostic rate of LEAD within the diabetic population, facilitating early detection and intervention to prevent the further progression of LEAD.

As stated in our manuscript, "Statistics indicate that over 60% of LEAD patients may be asymptomatic, leading to early stages of the disease often being overlooked. Compared to non-diabetic LEAD patients, diabetic peripheral neuropathy may mask LEAD symptoms, making the diagnosis of LEADDP more challenging. Current guidelines recommend initial screening for LEAD based on patient interviews and clinical examinations, using the Fontaine or Rutherford scales for assessment. This diagnosis heavily relies on the expertise of specialists and examination conditions, and the diagnostic performance is not ideal." (line 49-56)

Due to a lack of specialized knowledge, diabetic patients may not pay attention to symptoms such as reduced skin temperature in the lower extremities or mild pain, leading them to conceal their medical history and miss timely specialist examinations. They might seek care for diabetes or its complications in endocrinology or other departments, rather than choosing vascular surgery or peripheral vascular disease specialties to address LEAD. This could further result in LEAD being overlooked, delaying treatment. Therefore, we aim to address this issue through our study.

Our model predicts the risk of LEAD at a specific moment by integrating the clinical data of patients, allowing for the identification of high-risk populations. To illustrate this point further, we would like to provide an example. Risk assessment for venous thromboembolism (VTE) has been widely implemented in clinical practice, where risk prediction incorporates not only laboratory test indicators but also factors such as comorbidities. While these comorbidities are associated with VTE, this does not imply that they cause VTE to occur. By evaluating the risk of VTE, high-risk patients can be identified early, enabling timely treatment to prevent further progression of the condition. Similarly, our study aims to identify high-risk LEAD patients to facilitate early detection and intervention.

In our research, we have incorporated machine learning algorithms. Compared to traditional risk prediction methods, machine learning algorithms are better equipped to handle complex datasets containing multiple variables and features. More importantly, the risk prediction models constructed using machine learning have the potential for full automation, thereby reducing the need for healthcare personnel intervention, lowering clinical workloads, and improving diagnostic efficiency. This prospect holds significant social value.

Therefore, we believe that this study holds significant clinical importance and public health value.

**Relationship Between Risk Factors and LEAD**

LEAD is part of a broader spectrum of vascular diseases, with complex pathophysiological mechanisms. Although numerous studies exist, the detailed pathogenic mechanisms remain unclear. In the diabetic population, LEAD may have unique mechanisms of onset. While these pathogenic mechanisms are causally related to LEAD, their ambiguity prevents us from incorporating them into our risk prediction for LEAD.

By analyzing the relationship between various risk factors and LEAD, we can identify comorbidities that share similar pathogenic mechanisms with LEAD. In our study, we found an association between hematologic diseases and LEAD. Although the common mechanisms underlying this relationship remain unclear, this finding may provide insights for future research. To illustrate this point, we can use the example of patients with polycythemia vera, who may simultaneously experience headaches and lower limb venous thrombosis. While there is an association between headaches and venous thrombosis, this does not imply a causal relationship. By analyzing these associations and conducting further research, we may uncover changes in the patients' blood cells and ultimately identify the mutated genes.

Such findings can help us better understand the pathophysiological processes of LEAD, laying the groundwork for developing more effective medical interventions. Considering these factors, we intend to dedicate a section of our discussion to exploring the potential associations between various risk factors and LEAD in the diabetic population.

**Comparison of This Study with Similar Research**

You pointed out that "the discussion section should include relevant studies on LEAD risk prediction models," and we agree with your observation. This supplementary section will be outlined in the following paragraph. While we believe that more attention is needed for research on LEAD in diabetic patients, it is important to note that some studies on LEAD risk prediction models already exist. Some of these studies have employed machine learning algorithms, while others have not. Compared to studies that did not use machine learning algorithms, the introduction of machine learning can effectively handle a greater number of variables and complex features, yielding better performance. Furthermore, by continuously updating data and retraining the models, the predictive capabilities can be further enhanced. Additionally, improving machine learning algorithms to establish diagnostic platforms can lower the barrier to use and enhance diagnostic efficiency, offering broader application prospects. Compared to similar studies that utilized machine learning algorithms, the risk prediction model constructed in this study demonstrates certain performance advantages. Moreover, to our knowledge, this is the first study to develop a LEAD risk prediction model using machine learning algorithms specifically for the diabetic population in China.

In response to this issue, we have made additions in the revised manuscript. In lines 258-263, we included the following content: "Previous studies have employed machine learning algorithms to develop risk prediction models for LEAD, achieving relatively good predictive performance. However, these studies did not specifically focus on diabetic patients, and thus could not capture the unique characteristics of LEAD occurrence in this population. The application of machine learning algorithms in LEAD risk prediction models specifically targeting diabetic patients remains relatively limited."

**Other changes:**

We have added two references in lines 437-444.

Finally, we would like to express our gratitude once again for your assistance in improving this study.
